# Hospital-Based Methadone and Buprenorphine Initiation Practices by Addiction Consult Services

**DOI:** 10.1001/jamanetworkopen.2025.26077

**Published:** 2025-08-07

**Authors:** Shawn M. Cohen, Elana Straus, David A. Fiellin, Jamie L. Pomeranz, Joji Suzuki, Jeanette M. Tetrault, Melissa B. Weimer, E. Jennifer Edelman, Paul J. Joudrey

**Affiliations:** 1 Program in Addiction Medicine, Yale School of Medicine, New Haven, Connecticut; 2 Department of Internal Medicine, Yale School of Medicine, New Haven, Connecticut; 3 Yale School of Medicine, New Haven, Connecticut; 4 Department of Emergency Medicine, Yale School of Medicine, New Haven, Connecticut; 5 Department of Health Policy and Management, Yale School of Public Health, New Haven, Connecticut; 6 Department of Occupational Therapy, University of Florida, Gainesville; 7 Department of Psychiatry, Brigham and Women’s Hospital, Boston, Massachusetts; 8 Harvard Medical School, Boston, Massachusetts; 9 Department of Social and Behavioral Sciences, Yale School of Public Health, New Haven, Connecticut; 10 Division of General Internal Medicine, Department of Medicine, University of Pittsburgh School of Medicine, Pittsburgh, Pennsylvania; 11 Center for Research on Health Care, Department of Medicine, University of Pittsburgh, Pittsburgh, Pennsylvania

## Abstract

**Importance:**

The emergence of fentanyl and other high-potency synthetic opioids (HPSOs) has not only been underlying overdose deaths, but has complicated initiation of methadone and buprenorphine for opioid use disorder (OUD) treatment, including in the hospital. In response, clinicians with addiction expertise have developed novel initiation practices, yet no studies have characterized initiation practices nationally.

**Objective:**

To assess the use of novel hospital-based practices for initiating methadone and buprenorphine.

**Design, Setting, and Participants:**

This cross-sectional survey study of directors of hospital-based addiction consult services (ACS) associated with addiction medicine and addiction psychiatry fellowships in hospitals in the US was conducted using a REDcap anonymous survey from October 2023 to April 2024.

**Exposure:**

Predefined methadone and buprenorphine initiation practices. Standard methadone initiation was defined as 40 mg oral maximum on day 1 with up-titration of 5 to 10 mg every 3 days. Rapid methadone initiation was defined as any initiation regimen more rapid than standard. Buprenorphine initiation practices included low dose, high dose, traditional, and rescue.

**Main Outcomes and Measures:**

The primary outcome was the proportion of ACS directors using predefined methadone and buprenorphine initiation practices. Perceived impact of the drug supply on methadone and buprenorphine initiation was assessed through a 5-point Likert scale ranging from strongly disagree to strongly agree. Typical selection of buprenorphine initiation practices was assessed using 7 case-based scenarios intended to represent common hospital scenarios.

**Results:**

Among 80 consult services, 58 directors (72.5%; median [IQR] age, 41 [38-50] years; 27 of 57 [47.3%] women) completed surveys, one of which was partially completed. Of 57 ACS directors, specialties included addiction medicine (41 respondents [71.9%]), addiction psychiatry (11 respondents [19.3%]), general or consult liaison psychiatry (11 respondents [19.3%]), and toxicology (2 respondents [3.5%]). Among those who reported initiating methadone (47 of 58 respondents [81.0%]), 33 (70.2%) agreed that HPSOs changed their methadone initiation practices. Of 46 respondents, 40 (87.0%) reported rapid initiation of methadone, and of those, 26 (65.0%) reported using rapid initiation for more than 50% of initiations. Full-agonist opioids were used by 31 of 46 ACS directors (67.4%) to treat withdrawal during methadone initiation. Of 58 respondents, 54 (93.1%) agreed that HPSOs changed their buprenorphine initiation practices. All 58 ACS directors reported that their initiation practices offered buprenorphine initiation, including 53 of 57 (92.9%) offering low dose, 50 of 57 (87.7%) offering traditional, 43 of 57 (75.4%) offering high dose , and 20 of 57 (35.1%) offering rescue. For 7 clinical cases provided, low-dose initiation was the most commonly endorsed method of buprenorphine initiation, except in the case of a person presenting in significant withdrawal 2 days after last fentanyl use.

**Conclusions and Relevance:**

The findings of this survey study of hospital-based academic ACS directors suggest that methadone and buprenorphine initiation has adapted to a shifting opioid supply, often outpacing research and changes in clinical guidelines.

## Key Points

**Question:**

How commonly are hospital-based addiction clinicians using novel methadone and buprenorphine initiation practices to address challenges brought by fentanyl and high-potency synthetic opioid use?

**Findings:**

In this national, cross-sectional survey study of 58 addiction fellowship–associated hospital consult service directors, practices including rapid methadone initiation, use of full-agonist opioids for opioid withdrawal, and use of low- and high-dose buprenorphine initiation were widespread.

**Meaning:**

These findings suggest that among hospital-based academic addiction consult directors, methadone and buprenorphine initiation in the hospital setting is adapting to a shifting opioid supply, often outpacing advances in research and clinical guidelines.

## Teaser

This survey study assesses hospital-based addiction consult services’ use of novel practices for initiating methadone and buprenorphine for opioid use disorder.

## Introduction

The emergence of high-potency synthetic opioids (HPSO), such as fentanyl and fentanyl analogues, has led to an overdose crisis in the US. Methadone and buprenorphine are the most effective medications for opioid use disorder (MOUD), reducing overdoses and opioid-related mortality by up to 50%.^1^ To reduce overdose deaths, timely initiation and retention of methadone and buprenorphine are critical.

While the drug supply has changed, clinical guidelines about methadone and buprenorphine initiation were based on research when heroin and prescription opioids dominated the opioid supply.^2,3^ Standard guidance about methadone initiation begins with 30 to 40 mg taken orally followed by up-titration every 3 to 5 days.^2^ Guidance for the initiation of the partial opioid agonist buprenorphine begins with repeated doses of 2 to 4 mg; this begins only after opioid withdrawal symptoms emerge to decrease the risk of precipitated opioid withdrawal, an abrupt worsening of withdrawal symptoms due to buprenorphine displacing full opioid agonists on the opioid receptors.

However, evidence suggests HPSOs complicate methadone and buprenorphine initiation due to their high affinity at the μ-opioid receptor, prolonged clearance due to lipophilicity, and creation of high tolerance.^4,5,6,7,8^ For methadone initiation, the high tolerance induced by HPSOs means the standard methadone approach may inadequately treat withdrawal, reducing retention early in treatment.^9^ For buprenorphine initiation, HPSOs are thought to lead to more challenging initiations and risk for precipitated opioid withdrawal.^7,10,11,12^

Initiation of methadone and buprenorphine in the hospital setting has proven benefits.^13,14^ With the ability to closely monitor patients and adequately address symptoms and complications, hospital-based addiction consult services (ACS) are at the forefront of innovations seeking to overcome methadone and buprenorphine initiation barriers fueled by HPSOs. ACS have described rapid (ie, faster dose escalation compared with standard initiation) methadone initiation protocols, and have found them to be feasible and effective.^15,16,17,18^ Hospital ACS adapted buprenorphine initiations through several novel approaches,^19^ namely, the use of higher initiation doses (high-dose initiation)^20^ and the use of initially low but gradually increasing buprenorphine doses while overlapping full opioid agonists (low-dose initiation).^21^ Evidence, including the data synthetized by ACS, led to the American Society of Addiction Medicine (ASAM) releasing an expert-based guidance document addressing the adaptation of buprenorphine initiations to HPSOs.^19^

Despite reports of novel methadone and buprenorphine initiation practices, there remains a dearth of evidence describing the practices that ACS are using. We examined academic ACS directors’ self-report of perceptions of HPSOs and hospital-based methadone and buprenorphine initiation practices nationally.

## Methods

### Study Setting and Data Sources

This survey study was deemed exempt from full review by the Yale University institutional review board. Reporting follows the American Association for Public Opinion Research (AAPOR) reporting guideline.^22^ From October 2023 to April 2024, we surveyed ACS directors associated with addiction fellowships about their current practices for hospital-based methadone and buprenorphine initiation from the 50 US states; Washington, DC; and Puerto Rico.

We used public databases^23,24^ to identify Accreditation Council of Graduate Medical Education (ACGME)–accredited addiction medicine fellowships (96 fellowships) and addiction psychiatry fellowships (54 fellowships) and lists of fellowship directors from the American College of Academic Addiction Medicine and the American Academy of Addiction Psychiatry. We emailed program directors to identify the directors of the ACS associated with their fellowship program.

### Study Sample and Recruitment

Potential respondents were directors of hospital-based ACS affiliated with ACGME-accredited addiction medicine and addiction psychiatry fellowship programs. We chose this group because it is geographically diverse and likely to lead in methadone and buprenorphine initiation practice and educating addiction specialists. If more than 1 ACS was associated with a program, each service was included that provided services at distinct sites.

Eligible respondents received a unique link to a self-administered online survey. Codirectors were sent a joint email with a unique survey link that could only be completed once to ensure no single ACS was overrepresented. We sent potential respondents up to 3 reminders. Respondents received a $50 electronic gift card for participation. Informed consent was obtained electronically prior to the start of the survey.

### Survey Development

The survey (eAppendix in Supplement 1) was based on a prior study assessing practice variation in low-dose buprenorphine initiation,^25^ literature and recent ASAM Clinical Consensus Statement,^19^ and the research team’s expert opinion. We examined the face validity of the survey through pilot testing with addiction medicine and addiction psychiatry clinicians. Case-based scenarios were refined using one-on-one cognitive interviews,^26^ a structured interview method used for eliciting understanding of the questions and thought process to ensure consistent interpretation. Cognitive interviews were conducted over video conference with a geographically diverse group of 3 addiction medicine and 3 addiction psychiatry specialists; after each interview, the cases were iteratively refined.

The survey included definitions for each methadone and buprenorphine initiation method. We defined standard methadone initiation based on 2021 Substance Abuse and Mental Health Services Administration guidance^2^ as 40 mg maximum on day 1 with up-titration of 5 to 10 mg every 3 days. Rapid methadone initiation was defined as any initiation regimen more rapid than the standard initiation (using higher doses or shorter duration between dose escalations).

We defined 3 buprenorphine initiation practices based on ASAM Clinical Considerations.^19^ (1) Traditional initiation starts with a first dose of 2 to 4 mg of buprenorphine after reaching moderate withdrawal. (2) High-dose initiation (ie, macrodosing) starts with the first dose (8-16 mg) of buprenorphine after reaching moderate withdrawal. (3) Low-dose initiation (ie, overlapping or microdosing) starts with low doses of buprenorphine (eg, 0.5 mg sublingual), which gradually increases while continuing full opioid agonists. We defined a fourth option, buprenorphine rescue (ie, buprenorphine administration after intentional naloxone-precipitated withdrawal) based on recent case reports.^27,28^

### Survey Variables

Study data were collected and managed using REDCap.^29,30^ We assessed perceived impact of the drug supply on methadone and buprenorphine initiation through a 5-point Likert scale ranging from strongly disagree to strongly agree.

We assessed if methadone and buprenorphine initiation were offered. If buprenorphine initiation was offered, we assessed use of long-acting injectable formulations prior to discharge.

Regarding methadone initiation, we assessed the use of rapid initiation, additional use of full-agonist opioids to treat continued withdrawal, and other adjunctive medications to treat withdrawal (eg, clonidine). For services that used rapid methadone initiation, we assessed the proportion of initiations using rapid initiation in the last 2 weeks (1%-100% in 10% increments) as well as the clinical situations in which rapid initiation was used (ie, regular fentanyl use, pregnancy, prior premature discharge against medical advice, concomitant acute pain and OUD, recently discontinued methadone within last month, persistent withdrawal despite standard protocol, or other). We asked about the typical dose of methadone on days 1 to 5 of a rapid initiation through free-text response. For services that did not initiate methadone, we queried their approach to management of methadone prescribed as an outpatient.

Regarding buprenorphine initiation, we used multiple choice questions assessing use of each of the buprenorphine initiation practices defined previously and the proportion of initiations using each practice in the last 2 weeks (1%-100% in 10% increments). Low-dose initiation was further characterized by the formulations of buprenorphine used (split films or tabs, buccal, transdermal, intravenous, and other) and the length of a typical low-dose initiation.

We assessed typical selection of buprenorphine initiations practices using 7 case-based scenarios intended to represent common hospital scenarios. All cases pertained to hospitalized patients with severe OUD not interested in in other forms of MOUD. In brief the cases included: (1) a person using fentanyl with last use hours ago and short expected hospital length of stay, (2) a person using fentanyl with last use hours ago and long expected hospital length of stay, (3) a person using fentanyl in acute opioid withdrawal with last use days ago, (4) a person taking long-term opioids for pain now with severe OUD, (5) a person using fentanyl now with prolonged hospitalization taking opioids for pain, (6) a person with OUD in remission treated methadone with a relative contraindication to continuing methadone (loss of transportation access), and (7) a person with OUD in remission treated methadone with an absolute contraindication to continuing methadone (Torsades de Pointes and prolonged corrected QT interval).

Participants self-reported their demographic information and consult service characteristics. Race and ethnicity were self-identified and collected as required by the funder. Race categories were defined by Office of Management and Budget 1997 Revisions to the Standards for the Classification of Federal Data on Race and Ethnicity, including American Indian or Alaskan Native, Asian, Black or African American, Native Hawaiian or Other Pacific Islander, White, other (free-text response), and prefer not to answer. Ethnicity categories included Hispanic or Latino, not Hispanic or Latino, and prefer not to answer. Respondents could identify as more than 1 category.

### Statistical Analysis

For categorical variables, we used frequencies to describe the data. For continuous variables, we used median and IQR. Analyses were conducted in STATA 18 (StataCorp).^31^

## Results

We identified 83 ACS directors from 80 unique ACS associated with 77 ACGME-accredited addiction medicine and addiction psychiatry fellowships. Three ACS with 2 codirectors were sent a joint invitation. Of the 80 survey invitations, 58 directors responded (72.5% response rate). One survey was partially completed and was included in perceptions analysis; this survey provided partial information about medication initiation (only whether they initiated methadone, buprenorphine, and/or naltrexone but not specific practices used) and was not included in analysis of respondent characteristics.

### Respondent Characteristics

Of 57 ACS respondents (median [IQR] age, 41 [38-50] years), 41 (71.9%) were in addiction medicine, 11 (19.3%) were in addiction psychiatry, 11 (19.3%) were in general or consult liaison psychiatry, and 2 (3.5%) were in toxicology (Table). The services were associated with addiction medicine fellowships (48 respondents [84.2%]), followed by general or consult liaison psychiatry fellowships (14 respondents [24.6%]), addiction psychiatry fellowships (13 respondents [22.8%]), medical toxicology fellowships (6 respondents [10.5%]), and pain fellowships (2 respondents [3.5%]). The services saw a median (IQR) of 11 (7-20) new consults for patients with OUD per week. ACS were predominantly in urban hospitals (47 respondents [82.5%]) and located in the Northeast (24 respondents [42.1%]) and West (18 respondents [31.6%]), South (8 respondents [14.0%]), and Midwest (7 respondents [12.3%]).

**Table.** Respondent Characteristics

Characteristics	Respondents, No. (%) (N = 57)^a^
Addiction consult service characteristics	
Primary specialty of consult service^b^	
Addiction medicine	41 (71.9)
Addiction psychiatry	11 (19.3)
General or consult liaison psychiatry	11 (19.3)
Pain	0
Toxicology	2 (3.5)
Specialty of associated fellowship^b^	
Addiction medicine	48 (84.2)
Addiction psychiatry	13 (22.8)
General or consult liaison psychiatry	14 (24.6)
Pain	2 (3.5)
Toxicology	6 (10.5)
Hospital type	
Private, for profit	3 (5.2)
Private, not for profit	26 (44.8)
Public	23 (39.7)
US Department of Veterans Affairs	3 (5.2)
Other^c^	2 (3.5)
Hospital setting	
Rural	3 (5.3)
Suburban	7 (12.1)
Urban	47 (81.0)
Hospital size	
No. of hospitals with with data	56
No. of beds, median (IQR)^d^	588 (399-800)
New consults for OUD per wk	
No. with data	56
Median (IQR)	11 (7-20)
Hospital region	
Northeast	24 (41.3)
Midwest	7 (12.3)
South	8 (14.0)
West	18 (31.6)
Consult director characteristics	
Age, median (IQR), y	41 (38-50)
Race^b^	
American Indian or Alaska Native	0
Asian	7 (12.3)
Black	3 (5.3)
Native Hawaiian and other Pacific Islander	0
White	38 (66.7)
Prefer not to answer	6 (10.5)
Other^e^	10 (17.5)
Ethnicity	
Hispanic or Latino(a)	6 (10.5)
Not Hispanic or Latino(a)	47 (82.5)
Prefer not to answer	4 (7.0)
Gender	
Woman	27 (47.4)
Man	26 (45.6)
Nonbinary	1 (1.8)
Prefer not to answer	3 (5.3)
Clinician type	
MD or DO	56 (98.2)
Advanced practice provider (APRN or PA)	1 (1.8)
Residency completed^b^	
Emergency medicine	7 (12.3)
Family medicine	5 (8.6)
Internal medicine	25 (43.1)
Obstetrics and gynecology	0
Pediatrics	1 (1.8)
Preventative medicine	1 (1.8)
Psychiatry	21 (36.2)
Toxicology	1 (1.8)
Addiction board certifications, No./total No. (%)^f^	
Addiction medicine	42/56 (75.0)
Addiction psychiatry	5/56 (9.0)
Both	3/56 (5.4)
Neither	6/56 (10.7)
Years of medical practice, median (IQR)	10 (5-15)
Years of addiction practice, median (IQR)	6 (5-10)
Experience working at an opioid treatment program	37 (63.8)
Days on consult service per mo, median (IQR)	10 (5-20)

Abbreviations: APRN, advanced practice registered nurse; DO, doctor of osteopathic medicine; MD, medical doctor; PA, physician associate.

^a^ One of 58 respondents did not complete the characteristic question; all percentages were calculated out of 57.

^b^ More than 1 option could be selected.

^c^ Included state university–affiliated academic medical center and teaching hospital.

^d^ One answer of greater than 700 was calculated as 700 for analyses.

^e^ Other race allowed free-text responses and included Hispanic, Hispanic or Latino, Latine, Latino, Human, Middle Eastern, and 4 blank responses.

^f^ Only asked to MD and DOs.

 Of 57 respondents, 47 (82.5%) self-identified their ethnicity as not Hispanic or Latino, and 6 (10.5%) identified as Hispanic or Latino; for self-reported race, 7 respondents (12.3%) identified as Asian, 3 (5.3%) as Black or African American, 38 (66.7%) as White, and 10 (17.5) as another race, with 6 (10.5%) preferring not to answer. Respondents were split between women (27 respondents [47.3%]) and men (26 respondents [45.6%]), with 1 respondent (1.8%) identifying as nonbinary. Most were physicians (56 respondents [98.2%]), of whom 50 (89.3%) were board certified in addiction medicine and/or psychiatry. Respondents practiced addiction medicine or psychiatry for a median (IQR) of 6 (5-10) years. Nearly two-thirds (37 respondents [64.9%]) had experience working at an opioid treatment program (OTP).

### Perceptions of the Drug Supply

**Figure 1.**
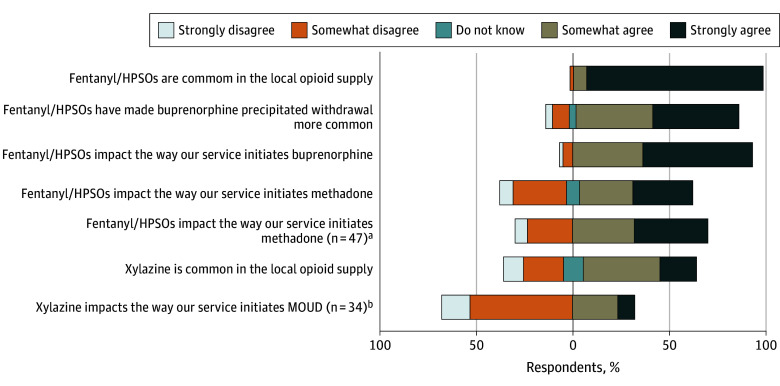
Hospital-Based Addiction Consult Service Directors’ Perception of the Impact of Opioid Supply on Methadone and Buprenorphine Initiation (n = 58 Directors) HPSO, High-potency synthetic opioids; MOUD, medication for opioid use disorder. ^a^Only including services that initiate methadone. ^b^Only including services that answer agree or strongly agree that xylazine is common.

Of 58 respondents, nearly all agreed (57 respondents [98.3%]) that HPSOs were common in the opioid supply, and 34 (58.6%) agreed xylazine was common (Figure 1). Of the 34 respondents who thought xylazine was common in the opioid supply, 11 (32.4%) agreed it impacted MOUD initiation.

### Methadone Initiation

For people with OUD not currently taking MOUD, 47 of 58 ACS directors (81.0%) offered methadone initiation. Of those, 33 (70.2%) agreed fentanyl changed methadone initiation.

**Figure 2.**
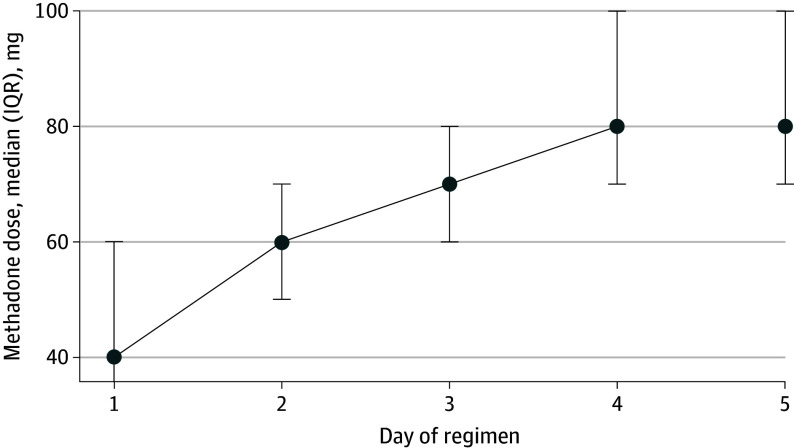
Rapid Methadone Up-Titration, Median Dose by Day of Regimen Among 39 Addiction Consult Services Of the 40 addiction consult services that used rapid methadone up titration, 1 respondent dropped because they listed 0 mg for all days of the rapid methadone up-titration regimen.

Rapid methadone initiation was used by 40 of 46 ACS directors (87.0%) that initiated methadone. A majority of those who used rapid initiation (26 of 40 ACS [65.0%]), reported using it for more than 50% of their methadone initiations, with 14 (35.0%) using rapid initiation for more than 90% of their methadone initiations. Typical reported methadone doses by day of rapid initiation regimen are displayed in Figure 2. Of the clinical situations provided, 76.1% of services that initiated methadone (35 of 46 ACS) reported using rapid initiation for persistent withdrawal, 67.4% (31 of 46 ACS) for regular fentanyl use, 58.7% (27 of 46 ACS) for pregnancy, 58.7% (27 of 46 ACS) for prior premature discharge, 58.7% (27 of 46 ACS) for concomitant pain and OUD, and 56.5% (26 of 46 ACS) for methadone discontinued within last month.

Adjunctive medications for the treatment of opioid withdrawal symptoms during methadone initiation were almost universally used (44 of 46 ACS [95.7%]). The most common were clonidine (36 of 46 ACS [78.3%]), hydroxyzine (34 of 46 ACS [73.9%]), full-agonist opioids such as oxycodone (31 of 46 ACS [67.4%]), loperamide (31 of 46 ACS [67.4%]), gabapentin (24 of 46 ACS [52.2%]), benzodiazepines (18 of 46 ACS [39.1%]), and ondansetron (5 of 46 ACS [10.9%]). Of 11 services that did not initiate methadone, all continued methadone if a patient was receiving methadone prior to hospitalization at the OTP-confirmed dose, and 5 (45.5%) offered to up-titrate the dose if clinically indicated.

### Buprenorphine Initiation

For people with OUD not currently taking MOUD, all 58 ACS offered buprenorphine initiation, including 21 (36.2%) additionally offering long-acting injectable buprenorphine during hospitalization. The majority of ACS directors (54 of 58 ACS directors [93.1%]) agreed that HPSOs changed the way they initiate buprenorphine, with 49 (84.5%) agreeing that HPSOs made precipitated opioid withdrawal with buprenorphine more common.

**Figure 3.**
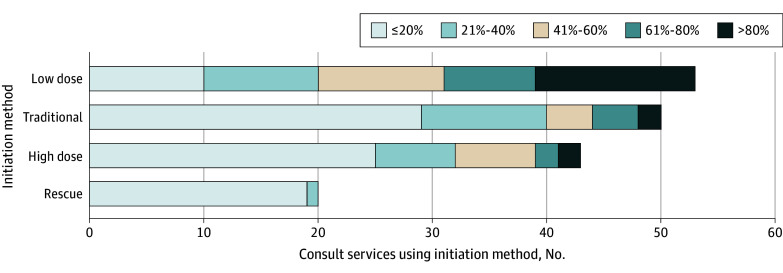
Frequency of Buprenorphine Initiation Practices Used by Addiction Consult Services (n = 57 Services) The frequency of the buprenorphine initiation practice used by addiction consult services (ACS) for each practice (low dose, traditional, high dose, and rescue) are displayed. Each bar represents the total number of ACS using that initiation practice and within each bar the frequency of use is stratified into quintiles. For example, for low dose initiation, a total of 53 of 57 ACS used this method and among those, 14 used it for more than 80% of their initiations, 8 used it for 61% to 80% of their initiations, 11 used it for 41% to 60% of their initiations, 10 used it for 21% to 40% of their initiations, and 10 used it for 1% to 20% of their initiations.

Low-dose buprenorphine initiation was offered by the highest proportion of ACS (53 of 57 ACS [92.9%]), followed by traditional (50 of 57 ACS [87.7%]), high dose (43 of 57 ACS [75.4%]), and buprenorphine rescue (20 of 57 ACS [35.1%]) (Figure 3). Of the 53 ACS that used low-dose initiation, the median (IQR) length of their initiation regimen was 5 (3-5) days. To administer doses of buprenorphine less than 2 mg, 28 (52.8%) used split films or tabs, 21 (39.6%) used buccal administration, 16 (30.2%) used transdermal administration, 8 (15.1%) used intravenous administration, 1 (1.9%) used the intravenous formulation of buprenorphine sublingually, and 1 (1.9%) used a formulation of buprenorphine-naloxone tablets available in the equivalent of 1-mg doses. Of these services, 14 (26.4%) used more than 1 of these formulations.

**Figure 4.**
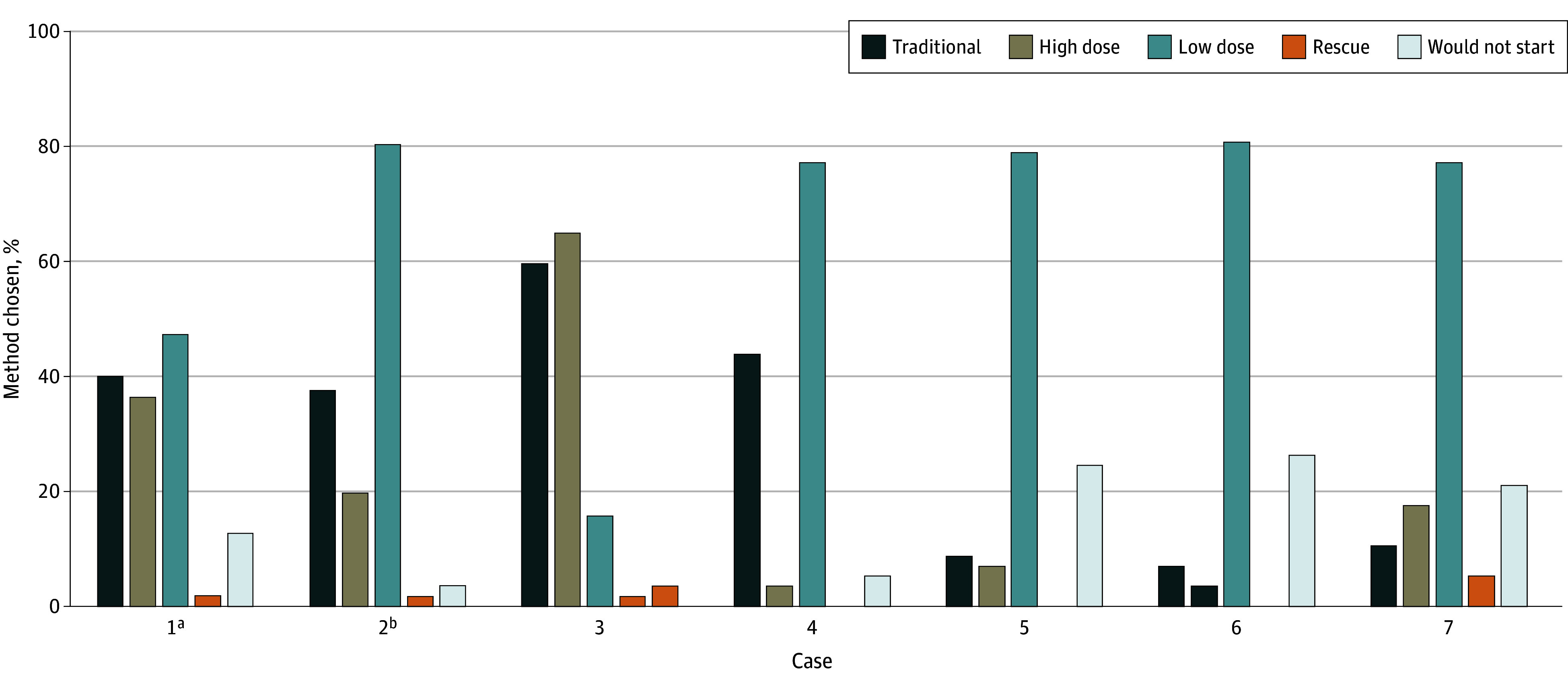
Buprenorphine Initiation Practice Chosen by Clinical Scenario (n = 57 Directors) Case 1: fentanyl use multiple times per day, last use being hours prior to admission, no current opioid withdrawal, and expected hospital discharge in 1 to 2 days. Case 2: fentanyl use multiple times per day, last use being hours prior to admission, no current opioid withdrawal, and expected hospital discharge in 1 week. Case 3: fentanyl use multiple times per day, last use being 2 days before admission, and Clinical Opiate Withdrawal Scale score of 12 with mydriasis and piloerection. Case 4: chronically prescribed 30 mg of immediate-release oxycodone every 6 hours for chronic pain from physician and currently diagnosed with severe opioid use disorder (OUD) in setting of using additional nonprescribed pharmaceutical oxycodone. Case 5: fentanyl use multiple times per day prior to admission, now admitted for 1 week with endocarditis and severe pain, and currently requiring opioid analgesics for acute pain. Case 6: OUD stable without substance use for years, receiving 100 mg of methadone in an outpatient setting, no longer can get to daily opioid treatment program, and last methadone dose was this morning. Case 7: OUD stable without substance use for years, receiving 100 mg of methadone, admitted to intensive care unit with Torsades, corrected QT-interval of 700 ms (electrolytes normal and no other QT-interval–prolonging medications), and last methadone dose this morning. ^a^Percentage calculated out of 55 (2 additional missing answers). ^b^Percentage calculated out of 56 (1 additional missing answer).

In the clinical cases presented (Figure 4), low-dose initiation was reported as the most frequently offered method of buprenorphine initiation in all cases other than one. A person presenting with significant opioid withdrawal (Clinical Opiate Withdrawal Scale score >12) with last fentanyl use 2 days prior was more commonly offered traditional or high-dose initiation.

## Discussion

To our knowledge, this is the first survey study to systematically characterize hospital methadone and buprenorphine initiation practices for OUD in a national sample. Our study generated several key findings. First, we found widespread agreement that HPSOs had changed their practices of initiating methadone and buprenorphine. Second, of those that initiated methadone, most (81%) offered rapid methadone initiation with variable maximum daily doses. When opioid withdrawal symptoms continued during methadone initiation, nearly all services (96%) offered ancillary medications, with a majority (67%) utilizing full-agonist opioids. Third, all services initiated buprenorphine, most commonly with low-dose initiation, followed closely by traditional and high-dose initiation. Across 7 clinical scenarios, low-dose buprenorphine initiation remained the most frequently offered practice except in the scenario of a patient presenting in significant opioid withdrawal 2 days after last fentanyl use in which traditional or high-dose initiation were preferred.

To our knowledge, this is the first study assessing hospital-based methadone initiation practices across different institutions. In the setting of higher opioid tolerances due to HPSOs and undertreated opioid withdrawal contributing to poor outcomes like premature hospital discharges,^32,33^ adequate dosing of methadone and control of withdrawal is critical. Hospitalization is an opportunity to quickly achieve an effective methadone dose in a monitored setting to maximize the potential for improved treatment retention^15,16,17,18^ and offers a setting to utilize short-acting opioids for withdrawal management.^34^ We found widespread and frequent use of rapid methadone initiations with short-acting opioids for continued withdrawal symptoms. Together, these results show that practice changes, in response to an evolving drug supply, have outpaced clinical guidelines and rigorous research in this area, and that research is needed to fully elucidate risks and benefits of each novel initiation practice. Despite this research gap, our results suggest collective consensus among ACS directors of the benefits of more assertive approaches in the hospital setting that outweigh concerns about the risks.

The impact of HPSOs on buprenorphine initiation is an area of ongoing research. There is widespread perception that buprenorphine-precipitated opioid withdrawal is increasingly common and that this is an underlying factor of buprenorphine initiation adaptations, which this study confirmed,^4,10,11^ yet hospital- and emergency department–based observational data has found widely variable rates of buprenorphine-precipitated opioid withdrawal ranging from 1%^6,35^ to more than 10%.^6,7,36^ Regardless of the true incidence, our study suggests that ACS are adopting novel buprenorphine initiation practices such as low-dose initiation. For hospitalized patients for whom full-agonist opioids can be administered, low-dose buprenorphine initiation offers several additional potential advantages: reducing need for opioid withdrawal, expanding clinical situations in which buprenorphine can be initiated to include those in which opioids must be continued (ie, for acute pain or for a person currently treated with methadone),^21^ and being the novel method with the most robust observational hospital-based data.^37,38,39,40^ Despite its common use, the specifics of low-dose initiation regimens, such as length of regimen and buprenorphine formulation, remain highly variable.^21,25,37,38,39,40^ Future research should examine the comparative effectiveness of the different low-dose initiation regimens.

While there was widespread use of novel initiation practices, there remained variability in details of these practices. Some variability in initiation should be expected given that ACS individualize care to adapt to the local drug supply^41^ and patient preference, yet, the amount of variability may reflect inconsistent dissemination and implementation of new practices, variable clinician comfort with practices prior to incorporation into guidelines, contradictory hospital and pharmacy guidance, and lack of comparative effectiveness data. As the drug supply continues to shift, as evidenced by new adulterants such as xylazine,^42^ guidelines are often not able adapt or be implemented quickly enough due to a lack of established evidence base and the known time lag in implementation of guidelines.^43^ Learning about health care systems and community-participatory research and leveraging the experience of the community of people who use drugs and community-based clinicians to understand and adapt to changes in the drug supply, such as through the Delphi Method,^44^ offers potential avenues to respond more quickly.^5^

An additional finding was the lack of self-identified racial and ethnic diversity among ACS directors. Despite overdose rates rising more quickly in racially and ethnically minoritized populations, particularly among Black, Hispanic, and American Indian or Alaska Native people,^45,46,47^ only 5% of our sample self-identified as Black, 10% identified as Hispanic, and none identified as American Indian or Alaska Native; this highlights the need to develop and invest in strategies to diversify and retain a workforce that reflects the communities they serve.^48^

### Limitations

This study has limitations. First, our study was limited to academic addiction fellowship–associated ACS in predominantly urban settings, and results may not generalize to services in other settings. Second, the study captured self-report of current practice, which may differ from actual practice. Third, we surveyed about adaptation in methadone and buprenorphine initiation based on published practices and thus may not have captured unpublished practices. Fourth, although our definitions (ie, rapid methadone initiation and low-dose buprenorphine initiation) were based on the literature and were provided throughout the survey, respondents may use different definitions to describe their practice, which may not have been adequately captured.

## Conclusions

In this survey study of hospital-based ACS directors, we found widespread agreement that HPSOs impact methadone and buprenorphine initiation and widespread use of novel initiation practices in response. Rapid methadone initiation and adjunctive use of full-agonist opioids to treat additional withdrawal were common. Low-dose initiation of buprenorphine was the most common buprenorphine initiation method used. Future research should assess the safety and effectiveness of these adaptations on patient outcomes and use community-partnered research to aid in implementation of new adaptations in response to a continuously shifting drug supply. Leveraging community expertise to generate time-sensitive guidance, such as through the Delphi Method, may serve as an important bridge to adapt clinical care more rapidly.

## Notes

Supplement 1. **eAppendix.** Hospital-Based Opioid Agonist Treatment Initiation Survey

Supplement 2.
Data Sharing Statement
