# Supplementary material for: Hospital-Based Methadone and Buprenorphine Initiation Practices by Addiction Consult Services
Source: JAMA Netw Open. 2025 Aug 7;8(8):e2526077. doi: 10.1001/jamanetworkopen.2025.26077 (PMC12332638; doi:10.1001/jamanetworkopen.2025.26077)
Supplement: Supplement 1. — eAppendix. Hospital-Based Opioid Agonist Treatment Initiation Survey [file jamanetwopen-e2526077-s001.pdf]

Supplemental Online Content

Cohen S, Straus E, Fiellin DA, et al. Hospital-based methadone and buprenorphine initiation practices by addiction consult services. *JAMA Netw. Open*. 2025;8(8):e2526077. doi:10.1001/jamanetworkopen.2025.26077

eAppendix. Hospital-Based Opioid Agonist Treatment Initiation Survey

This supplemental material has been provided by the authors to give readers additional information about their work.

© 2025 Cohen S et al. *JAMA Network Open*.

eAppendix. Hospital-Based Opioid Agonist Treatment (OAT) Initiation Survey

Welcome to the Hospital-Based OAT Initiation Survey!

The purpose of this survey is to learn about your addiction consult services' experiences initiating methadone and buprenorphine in the hospital including the protocols you use. This survey should take about 20 minutes to complete and upon completion you will be compensated through email with a $50 e-gift card. Please answer each question to the best of your ability. If more than one answer applies, please select the answer that best captures your response. This study is being administered through Yale School of Medicine. Your participation is completely voluntary.

Your responses will be kept confidential, will only be shared in aggregate. The results of the study will be used to describe current practices and develop consensus on best practices to improve the care of people with opioid use disorder in the hospital. This an area of little but much needed research and standardization.

For more information please click here.

If you have any questions, please email Shawn Cohen MD athospitaloatstudy@yale.edu.

Section 1: General Questions

In a hospitalized patient not being treated with medication(s) for opioid use disorder prior to admission, which medications does your service start?

Do NOT include if you use the medication only as a taper for withdrawal management

- ☐ Buprenorphine
- ☐ Methadone
- ☐ Naltrexone
- ☐ None

Do you offer long acting injectable buprenorphine (sublocade and/or brixadi) prior to hospital discharge?

- ◯ Yes
- ◯ No

Do you offer naltrexone IM (vivitrol) for Opioid Use Disorder prior to hospital discharge?

- ◯ Yes
- ◯ No

Please rate your agreement with the following statements regarding your consult service's experience with fentanyl/high potency synthetic opioids (HPSO) HPSOs=fentanyl, fentanyl analogues, and other man-made opioids that are more potent than heroin

	Don't know	Strongly Disagree	Disagree	Agree	Strongly Agree
Fentanyl/HPSOs are common in the local opioid supply	◯	◯	◯	◯	◯
Fentanyl/HPSOs have made buprenorphine precipitated withdrawal more common	◯	◯	◯	◯	◯
Fentanyl/HPSOs impact the way our service initiates buprenorphine	◯	◯	◯	◯	◯
Fentanyl/HPSOs impact the way our service initiates methadone	◯	◯	◯	◯	◯

© 2025 Cohen S et al. *JAMA Network Open*.

Xylazine is common in the local opioid supply	○	○	○	○	○
Xylazine impacts the way our service initiates MOUD	○	○	○	○	○

25%

Progress

© 2025 Cohen S et al. *JAMA Network Open*.

Section 2: Methadone Initiation

Please rate your agreement with the following statements
You are being asked this question because you answered that you do NOT start methadone in the hospital

	Don't know/Not sure	Strongly Disagree	Disagree	Agree	Strongly Agree
It is illegal to start methadone in the hospital	◯	◯	◯	◯	◯
There are insufficient connections to continue methadone in the community	◯	◯	◯	◯	◯
Methadone is NOT effective treatment of OUD	◯	◯	◯	◯	◯
I do NOT have the expertise/experience to start methadone in the hospital	◯	◯	◯	◯	◯
My institution will NOT allow methadone to be started in the hospital	◯	◯	◯	◯	◯

What is your standard practice in managing a patient's outpatient methadone dose if there is no clinical contraindication to continuing the methadone?

- ◯ Confirm their dose with the OTP and then continue methadone at that dose
- ◯ Confirm their dose with the OTP and provide methadone up to a specific dose limit
- ◯ Provide all people on methadone a pre-specified dose in the hospital
- ◯ Discontinue methadone during hospitalization
- ◯ Other

What is your methadone continuation dose limit?

____
(mg)

What is the pre-specified methadone dose you provide to all patients on methadone?

____
(mg)

What is your practice in managing a patient's outpatient methadone dose?

____

Does your service ever uptitrate methadone more rapidly than the standard protocol?

Standard protocol:
Day 1 = first dose up to 30mg + 10mg available for continued withdrawal (max 40mg on day 1).
Day 2 and beyond = increase by 5-10mg every 3-5 days guided by withdrawal/cravings
(based on SAMHSA TIP 63)

- ◯ Yes
- ◯ No

© 2025 Cohen S et al. *JAMA Network Open*.

In your last 2 weeks working on the consult service, approximately what percent of methadone initiations your service did were more rapid than the standard protocol?

Standard protocol:
Day 1 = first dose up to 30mg + 10mg available for continued withdrawal (max 40mg on day 1).
Day 2 and beyond = increase by 5-10mg every 3-5 days guided by withdrawal/cravings
(based on SAMHSA TIP 63)

- ◯ < 10%
- ◯ 11-20%
- ◯ 21-30%
- ◯ 31-40%
- ◯ 41-50%
- ◯ 51-60%
- ◯ 61-70%
- ◯ 71-80%
- ◯ 81-90%
- ◯ >90%

In what clinical situations does your service uptitrate methadone more rapidly than the standard protocol?

- ☐ Regular fentanyl use
- ☐ Pregnancy
- ☐ Prior premature discharge (AMA)
- ☐ Concomitant acute pain and OUD
- ☐ Recently discontinued methadone (within last month)
- ☐ Persistent withdrawal despite standard protocol
- ☐ Other ______

Do you have a written rapid methadone uptitration protocol?

- ◯ Yes
- ◯ No

What is the maximum daily dose of methadone (mg) in your typical rapid methadone uptitration?
Including all standing doses and available PRN doses Day 1: ______ mg
Day 2: ______ mg
Day 3: ______ mg
Day 4: ______ mg
Day 5: ______ mg

During rapid methadone uptitration, how often have patients experienced opioid toxicity from the methadone requiring pausing uptitration?

opioid toxicity=respiratory depression and/or somnolence

- ◯ Never
- ◯ Rarely
- ◯ Occasionally
- ◯ Often
- ◯ Always

During rapid methadone uptitration, how often have patients experienced opioid toxicity from the methadone requiring naloxone reversal?

opioid toxicity=respiratory depression and/or somnolence

- ◯ Never
- ◯ Rarely
- ◯ Occasionally
- ◯ Often
- ◯ Always

Does your consult service typically use additional full agonist opioids (ie oxycodone, hydromorphone...) to treat opioid withdrawal while uptitrating methadone?

- ◯ Yes
- ◯ No

Does your consult service typically use other medications to treat opioid withdrawal while uptitrating methadone?

- ◯ Yes
- ◯ No

Which medications do you use for this?

- ☐ Clonidine
- ☐ Hydroxyzine
- ☐ Loperamide
- ☐ Gabapentin
- ☐ Benzodiazepines
- ☐ Other ______

© 2025 Cohen S et al. *JAMA Network Open*.

What is/are the biggest lesson(s) you learned while implementing rapid methadone uptitration?	__________________________________
How often is your service able to complete admission/enrollment in an opioid treatment program (OTP) during hospital admission?<br><br>OTP=outpatient methadone program<br><br>Admission/enrollment = Completion of OTP intake while hospitalized so the patient is an established patient at the OTP on discharge	◯ Always<br>◯ Very Often<br>◯ Sometimes<br>◯ Rarely<br>◯ Never
Does your service ever increase a patient's methadone dose while admitted to the hospital?	◯ Yes<br>◯ No
Is there at least one local opioid treatment program (OTP) that will continue methadone at the dose uptitrated to in the hospital?	◯ Yes they continue the dose we uptitrate to<br>◯ No they restart the patient as a new initiation<br>◯ Don't know/not sure
Has your service ever experienced hospital discharge delays related to connecting patients with methadone?	◯ Yes<br>◯ No
In what situations have you has your service experienced hospital discharge delays related to connecting patients with methadone?	☐ Weekend discharges<br>☐ Holiday discharges<br>☐ Opioid Treatment Program (OTP) wait lists<br>☐ OTP admissions only on certain days<br>☐ Short term rehab/Skilled nursing facility discharges<br>☐ Other ______

50%

Progress

© 2025 Cohen S et al. *JAMA Network Open*.

Section 3: Buprenorphine Initiation

Please rate your agreement with the following statements
You are being asked this question because you answered that you do NOT start buprenorphine in the hospital

	Don't know/Not Sure	Strongly Disagree	Disagree	Agree	Strongly Agree
It is illegal to start buprenorphine in the hospital	○	○	○	○	○
There are insufficient connections to continue buprenorphine in the community	○	○	○	○	○
Buprenorphine is NOT effective treatment of OUD	○	○	○	○	○
I do NOT have the expertise/experience to start buprenorphine	○	○	○	○	○
My institution will not allow buprenorphine to be started in the hospital	○	○	○	○	○

Methods of Buprenorphine Initiation In the following section you will be asked about which methods of buprenorphine initiation you use in the hospital. We describe 4 different potential strategies

1) Traditional initiation

- Begins with a period of opioid abstinence
- When mild/moderate symptoms of withdrawal are present, 2-4mg buprenorphine is given
- Additional doses given based on continued withdrawal symptoms
- FDA recommended maximum day 1 dose = 8mg, but the dose can be higher if the benefit outweighs the risk. Per ASAM National Practice Guideline For the Treatment of Opioid Use Disorder (1) and SAMHSA TIP 63 (2)

2) High Dose initiation ("macrodosing")

- Begins with a period of opioid abstinence
- When withdrawal symptoms are present, 8mg-16mg of buprenorphine is given
- Additional doses are repeated up to a total of 16-32mg buprenorphine on day 1 to rapidly reduce withdrawal symptoms. Per ASAM Clinical Considerations: Buprenorphine Treatment of Opioid Use Disorder for Individuals Using High-potency Synthetic Opioids (3)

3) Low Dose/Overlapping Initiation ("microdosing")

- Full opioid agonists are continued
- Buprenorphine is started at very low doses (often 0.5mg) and uptitrated slowly over days
- Mechanism - Buprenorphine only minimally displaces the full agonist with each gradually increasing dose to avoid the need for withdrawal and minimize the risk of precipitated opioid withdrawal

- Different protocols use different formulations (sublingual, buccal, IV, or transdermal) for low doses of buprenorphine

© 2025 Cohen S et al. *JAMA Network Open*.

projectredcap.org

- Most commonly used when full agonists cannot be stopped (acute pain, transitioning from methadone) Example regimen

Day Buprenorphine dose Full Agonist
1 0.5mg sublingual every 3 hours Continue full dose
2 1mg sublingual every 3 hours Continue full dose
3 8-16mg sublingual and additional as needed STOP after 1st dose

Per ASAM Clinical Considerations: Buprenorphine Treatment of Opioid Use Disorder for Individuals Using High-potency Synthetic Opioids (3) and Low Dose Initiation of Buprenorphine: A Narrative Review and Practical Approach (4)

4) Buprenorphine rescue from opioid antagonist precipitated withdrawal

- Precipitated withdrawal is first intentionally caused by use of an opioid antagonist (naloxone or naltrexone)

- Buprenorphine is then given to relieve withdrawal symptoms and start treatment.
Example Regimen
Adminisiter naloxone intranasally
When withdrawal symptoms are severe give 24mg buprenorphine as a single dose

Per Rapid Transition to Buprenorphine in a Patient With Methadone-Related QTc Interval Prolongation (5) and Enhancing Patient Choice: Using Self-administered Intranasal Naloxone for Novel Rapid Buprenorphine Initiation (6)

Click here for references

Please answer the following questions about your service's buprenorphine initiation methods

Traditional = Wait for opioid withdrawal then give 2-4mg buprenoprhine and repeat dose to effect
High Dose = Wait for opioid withdrawal then give 8-16+mg buprenoprhine and repeat dose to effect
Low dose/overlapping = Give gradually increasing buprenorphine doses over days while continuing full agonist opioids
Buprenorphine rescue = Give naloxone and after precipitating withdrawal give buprenoprhine to improve symptoms

Does your service use this method? In your past 2 weeks on service, what percent of initiations used this method? Does your service have a protocol for this method?

Traditional Initiation ______ ______ ______
High Dose Initiation ______ ______ ______
Low Dose/Overlapping Initiation ______ ______ ______
Buprenorphine rescue from opioid antagonist precipitated withdrawal ______ ______ ______

In the following 7 clinical scenarios, which of the following buprenorphine initiations method would you offer?

Traditional = Wait for opioid withdrawal then give 2-4mg buprenorphine and repeat dose to effect
High Dose = Wait for opioid withdrawal then give 8-16+mg buprenorphine and repeat dose to effect
Low dose/overlapping = Give gradually increasing buprenorphine doses over days while continuing full agonist opioids
Buprenorphine rescue (rescue) = Give naloxone and after precipitating withdrawal give buprenorphine to improve symptoms

All patients are currently hospitalized, have severe OUD, and are not interested in other forms of MOUD (methadone or naltrexone). The buprenorphine initiation can be completed at the moment presented or later during the hospital course. You can choose more than one method. If not otherwise specified, assume the patient will remain in the hospital as long as needed to complete the chosen buprenorphine initiation method(s). You can use full opioid agonists as a part of any of the initiation methods.

© 2025 Cohen S et al. *JAMA Network Open*.

Multiple times per day fentanyl use, last use hours prior to admission, no current opioid withdrawal. Expected hospital discharge in 1-2 days ______
Multiple times per day fentanyl use, last use hours prior to admission, no current opioid withdrawal. Expected hospital discharge in 1 week ______
Multiple times per day fentanyl use, last use 2 days before admission, COWS 12 with mydriasis and piloerection ______
Chronically receiving prescribed oxycodone IR 30mg every 6hrs for chronic pain from physician, now diagnosed with severe OUD in setting of using additional nonprescribed pharmaceutical oxycodone ______
Multiple times per day fentanyl use prior to admission, now admitted x 1 week with endocarditis and severe pain, currently requiring opioid analgesics for acute pain ______
OUD stable without substance use x years on 100mg methadone in outpatient setting, no longer can get to OTP daily, last methadone dose this AM ______
OUD stable without substance use x years on 100mg methadone, admitted to ICU with torsades, QTc 700 (electrolytes normal, no other QT prolonging medications), last methadone dose this AM ______

What additional information would you like us to know about your management in the above cases?

____

In your typical low dose/overlapping initiation, what buprenorphine formulation do you use for the < 2mg doses of buprenorphine?

typical=most commonly used low dose/overlapping method/protocol

- ☐ Split films/tabs
- ☐ Buccal
- ☐ Transdermal (patch)
- ☐ Intravenous (IV)
- ☐ Other ______

How many days is your typical low dose/overlapping initiation?

typical=most commonly used low dose/overlapping method/protocol

____
(days)

While completing a low dose/overlapping initiation, what is your general practice in managing the full opioid agonists the patient is taking?

Assuming the patient does not have another indication for full opioid agonists (e.g. acute pain)

- ◯ Start tapering full agonist BEFORE STARTING the low dose initiation
- ◯ Start tapering full agonist WHEN STARTING the low dose initiation
- ◯ Start tapering full agonist DURING the low dose initiation process
- ◯ Start tapering full agonist AFTER COMPLETING the low dose initiation
- ◯ Abruptly stop full agonists when low dose initiation is complete
- ◯ Other ______

What is/are the biggest lesson(s) you learned while implementing different initiation approaches for buprenorphine?

____

75%

Progress

© 2025 Cohen S et al. *JAMA Network Open*.

Part 4: Demographics

What is your age (years)?

______________________ (years)

What race or races do you identify as?

Select all the apply

- ☐ American Indian or Alaskan Native
- ☐ Asian
- ☐ Black or African American
- ☐ Native Hawaiian and Other Pacific Islander
- ☐ White
- ☐ Other ______
- ☐ Prefer Not to Answer

Which of the following best represents your gender?

- ◯ Female
- ◯ Male
- ◯ Non-binary
- ◯ Trans-female
- ◯ Trans-male
- ◯ Prefer to self describe ______
- ◯ Prefer not to answer

What ethnicity do you identify as?

- ◯ Hispanic or Latino(a)
- ◯ Not Hispanic or Latino(a)
- ◯ Prefer Not to Answer

What type of clinician are you?

- ◯ MD/DO
- ◯ APP (APRN, PA, ...)
- ◯ Other ______

What specialty or specialties did you complete residency in?

- ☐ Emergency Medicine
- ☐ Family Medicine
- ☐ Internal Medicine
- ☐ OB GYN
- ☐ Pediatrics
- ☐ Preventative Medicine
- ☐ Psychiatry
- ☐ Other ______

Are you board certified or board eligible in addiction?

- ◯ Yes in Addiction Medicine
- ◯ Yes in Addiction Psychiatry
- ◯ Yes in both Addiction Medicine and Addiction Psychiatry
- ◯ No

How many years have you been practicing medicine as an independent clinician (attending physician or APP graduate)?

______________________ (years)

How many years have you been practicing addiction [medicine or psychiatry] as an independent clinician (attending physician or APP graduate)?

______________________ (years)

© 2025 Cohen S et al. *JAMA Network Open*.

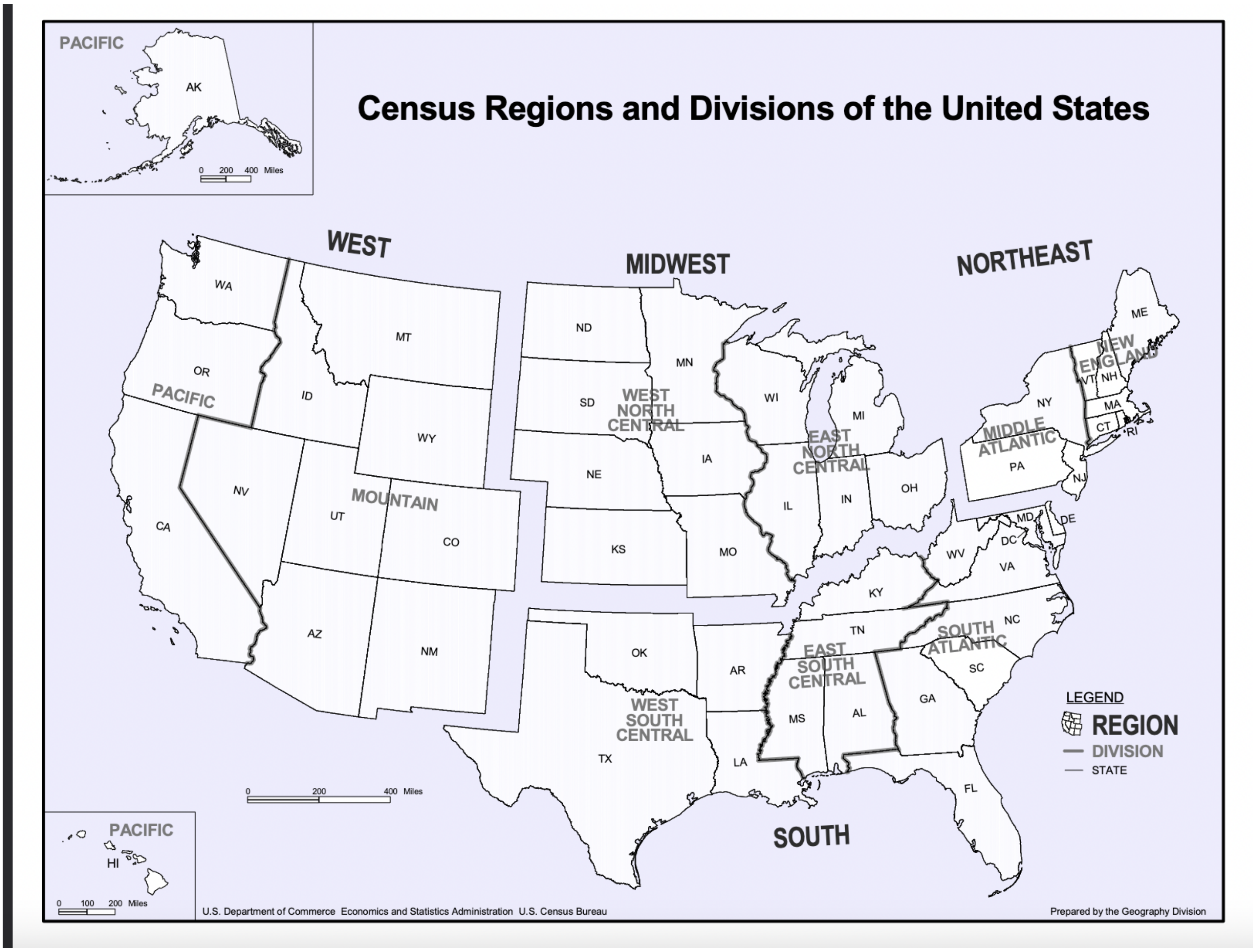

In what region of the United States do you practice?	◯ Northeast<br>◯ Midwest<br>◯ South<br>◯ West
Have you ever worked at an opioid treatment program?	◯ Yes<br>◯ No
On average, how days per month do you clinically work on the consult service?	______________________
What is the specialty of the fellowship(s) your consult service is associated with?	☐ Addiction Medicine<br>☐ Addiction Psychiatry<br>☐ Consult Liason Psychiatry<br>☐ Pain<br>☐ Toxicology<br>☐ Other ______

© 2025 Cohen S et al. *JAMA Network Open*.

What is the primary specialty of your consult service?	☐ Addiction Medicine<br>☐ Addiction Psychiatry<br>☐ General or Consult Liason Psychiatry<br>☐ Pain<br>☐ Toxicology<br>☐ Other ______
How would you classify the hospital type where your consult service is located?	◯ Private/for profit<br>◯ Private/non-profit<br>◯ Public<br>◯ Other ______
How would you classify the hospital setting where your consult service is located?	◯ Rural<br>◯ Suburban<br>◯ Urban
Approximately how many beds is the hospital where your consult service is located?	______________________________<br>(beds)
Approximately how many new consults for opioid use disorder does your service receive in a week?	______________________________

If you would like, please identify someone you consider an expert in hospital initiation of opioid agonist therapy for possible inclusion in a group to create best practices Name: ______ Email: ______

What additional comments do you have about hospital initiation of opioid agonist therapy?	_____________________________________

© 2025 Cohen S et al. *JAMA Network Open*.
