# Supplementary material for: Hospital-Based Methadone and Buprenorphine Initiation Practices by Addiction Consult Services
Source: JAMA Netw Open. 2025 Aug 7;8(8):e2526077. doi: 10.1001/jamanetworkopen.2025.26077 (PMC12332638; doi:10.1001/jamanetworkopen.2025.26077)
Supplement: Supplement 2. — Data Sharing Statement [file jamanetwopen-e2526077-s002.pdf]

Data Sharing Statement

Cohen. Hospital-Based Methadone and Buprenorphine Initiation Practices by Addiction Consult Services. *JAMA Netw Open*. Published August 07, 2025.
doi:10.1001/jamanetworkopen.2025.26077

Data
Data available: Yes
Data types: Deidentified participant data
How to access data: Data available by request to shawn.cohen@yale.edu
When available: With publication

Supporting Documents
Document types: None

Additional Information
Who can access the data: Anyone requesting data
Types of analyses: For any purpose
Mechanisms of data availability: With investigator support
